# Implications of Inaccurate Blood Pressure Measurement on Hypertension Prevalence

**DOI:** 10.1016/j.cjco.2024.10.011

**Published:** 2024-11-08

**Authors:** Alexander A. Leung, Swapnil Hiremath, Jeanne V.A. Williams, Ross T. Tsuyuki

**Affiliations:** a Department of Medicine, Cumming School of Medicine, University of Calgary, Calgary, Alberta, Canada; b Department of Medicine, Faculty of Medicine, University of Ottawa, Ottawa, Ontario, Canada; c Department of Community Health Sciences, Cumming School of Medicine, University of Calgary, Calgary, Alberta, Canada; d Department of Medicine, Division of Cardiology, Faculty of Medicine and Dentistry, University of Alberta, Calgary, Alberta, Canada

## Abstract

**Background:**

The cornerstone of the management of hypertension is accurate measurement of blood pressure (BP). A recent study showed that more than one-half of home BP devices sold in Canada have no evidence of validation for accuracy. The purpose of this study was to model the implications of inaccurate BP measurements on diagnosis and control of hypertension.

**Methods:**

We used data from the Canadian Health Measures Survey to model the effects of inaccurate BP devices by 5 or 10 mm Hg over or under the true BP value. Hypertension was defined as BP ≥ 140/90 mm Hg (or ≥130/80 mm Hg in those patients with diabetes).

**Results:**

If both systolic and diastolic BP were overestimated by 10 mm Hg, the prevalence of hypertension would falsely increase by 50% to 63%, potentially leading to overtreatment of approximately 3.5 million Canadians. Conversely, the impact of underestimation of BP appeared minimal, but mostly because of design limitations of our study.

**Conclusions:**

We found that overestimation of BP by only 10 mm Hg could lead to overtreatment of up to 3.5 million Canadians. Government should mandate the validation of BP devices before they can be sold; until then, clinicians and patients should be cautious in their selection of BP devices, using the Hypertension Canada Recommended Device Program to guide selection.

## Résumé

**Contexte:**

La pierre angulaire de la prise en charge de l'hypertension est la mesure précise de la pression artérielle (PA). Une étude récente a montré que plus de la moitié des appareils de mesure de la PA à domicile vendus au Canada n'ont pas fait l'objet d'une validation de leur précision. L'objectif de cette étude était de modéliser les implications des mesures inexactes de la PA sur le diagnostic et le contrôle de l'hypertension.

**Méthodes:**

Nous avons utilisé les données de l'Enquête canadienne sur les mesures de la santé (ECMS) pour modéliser les répercussions des dispositifs de mesure de la PA imprécis, de 5 ou 10 mmHg au-dessus ou au-dessous de la valeur réelle de la PA. L'hypertension a été définie comme une PA ≥ 140/90 mmHg (ou ≥130/80 mmHg chez les patients diabétiques).

**Résultats:**

Si la PA systolique et diastolique était surestimée de 10 mmHg, la prévalence de l'hypertension augmenterait à tort de 50 % à 63 %, ce qui pourrait conduire à un surtraitement d'environ 3.5 millions de Canadiens. Inversement, l'impact de la sous-estimation de la PA semble minime, mais principalement en raison des limites de la conception de notre étude.

**Conclusions:**

Nous avons constaté qu'une surestimation de la PA de seulement 10 mmHg pouvait entraîner un surtraitement chez 3.5 millions de Canadiens. Le gouvernement devrait rendre obligatoire la validation des appareils de mesure de la PA avant qu'ils ne soient vendus; d'ici là, les cliniciens et les patients devraient faire preuve de prudence dans leur choix d'appareils de mesure de la PA, en s'inspirant du programme d'appareils recommandés par Hypertension Canada pour guider leur sélection.

Elevated blood pressure (BP) is a risk factor for several adverse cardiovascular outcomes, and lowering BP has been shown in several randomized clinical trials (RCTs) to reduce this risk: in particular, stroke, heart failure, and cardiovascular (CV) death.^1^^,^^2^ The cornerstone of BP lowering relies on first measuring BP correctly, including adequate resting, and proper procedural aspects such as slow deflation and avoiding terminal digit preference.^3^ The development of oscillometric BP measurement devices has circumvented many of these measurement issues and has also allowed for measurement of multiple BP readings and their averaging, thus mimicking the practice in RCTs in real-world clinical practice as well as patient performed self-home BP measurements.^4^ Automated oscillometric or standardized BP measurements are now recommended by all major hypertension professional societies for diagnosis and management of hypertension.^5^ In addition, home BP monitoring in particular has been reported to be a strong predictor of CV morbidity and mortality, and meta-analyses support the use of home BP to be associated with a significantly lower achieved BP.^6^^,^^7^

In Canada, using the last Canadian Health Measures Survey (CHMS, 2007-2017), approximately 5.8 million people are living with hypertension, representing approximately 23% of the population.^8^ Globally, the use of home BP is reported to be approximately 30% to 70%, and in Canada to be just under 50%.^9^ Even as of 2017, most primary care providers from a survey relied on oscillometric BP devices, and almost 70% also used home BP measurements for guiding ongoing management.^10^

At the same time, issues of validation and accuracy remain a concern. Internationally, less than 15% of the BP measurement devices sold are validated.^11^ As a consequence, approximately 5 years ago, Hypertension Canada developed a reliable validated device listing so that accurate devices can be easily identified (https://hypertension.ca/healthcare-professionals/recommended-devices).^12^ However, from a recent Canadian survey, 55% of the home BP devices available for online sale in Canada were not listed on the Hypertension Canada device recommendation program (ie, have no evidence of validation).^13^ Although published reports of the prevalence of the magnitude of inaccuracy in measurement in BP devices in use varies from 24% to 69% based on criteria used for accuracy,^14^, ^15^, ^16^, ^17^ the actual impact of inaccurate BP measurements on rates of diagnosis and control for hypertension remains unknown. We used the data from the CHMS to model the public health effect of inaccurate BP measurement, whether by home or clinic BP devices, in the Canadian context.

## Material and Methods

### Data source

This study was conducted using the CHMS, which is an ongoing cross-sectional survey that collects self-reported demographic, socioeconomic, lifestyle, and health information through structured in-person interviews in the home, followed by direct physical measures of health (eg, BP, height, and weight) using standardized techniques at a mobile examination centre.^18^^,^^19^ Sampling for the CHMS was based on a complex multistage design so that nationally representative estimates of common medical conditions could be determined reliably.^19^^,^^20^ The sampling frame included community-dwelling Canadian residents aged 3 to 79 years (6 to 79 years in cycle 1; 3 to 79 years in cycles 2 to 6). Excluded from the survey were residents of the territories, as well as those who lived in remote regions, on reserves or in institutions, or who were full-time members of the Canadian Armed Forces, collectively representing approximately 3% of the Canadian population. For our study, the first (2007-2009), second (2009-2011), third (2012-2013), fourth (2014-2015), fifth (2016-2017), and sixth (2018-2019) cycles of the CHMS were included, representing all available cycles to date.^21^, ^22^, ^23^, ^24^, ^25^, ^26^

The cardinal strength of the CHMS is that it is the only ongoing national health survey in Canada that provides direct physical measurements (including BP), which are necessary to estimate the prevalence of hypertension in the population. The advantage of using a complex survey design is that it improves statistical efficiency, thus reducing costs, while still allowing for unbiased estimates to be calculated (with measurable sampling error). Correspondingly, Statistics Canada provides sampling weights to account for the unequal probabilities of participant selection, which are also adjusted for nonrespondents (to minimize nonresponse bias), and thus designed to provide accurate population projections (ie, in which each survey respondent provides inference about a larger number of people in the population).^19^^,^^20^ Accordingly, the CHMS has been used in numerous studies to describe the epidemiology of hypertension and its related risk factors in Canada.^8^^,^^27^, ^28^, ^29^, ^30^, ^31^, ^32^, ^33^ Common to all complex survey data, some of the disadvantages of the CHMS include more complicated approaches to statistical analysis (compared with simple random sampling), so that variance estimation must be based on linearization techniques or replication methods; the latter is recommended for the CHMS with bootstrapping and was used in the current study.^21^, ^22^, ^23^, ^24^, ^25^, ^26^^,^^34^^,^^35^

### Measures and definitions

#### Hypertension

For our current study, we focused on survey respondents who were aged 20 to 79 years, as representative of Canadian adults. Systolic blood pressure (SBP) and diastolic blood pressure (DBP) measurements were recorded in a standardized manner using an automated office BP technique with BpTRU BPM-200 and BPM-300 oscillometric devices (BpTRU Medical Devices Ltd, Coquitlam, British Columbia, Canada).^36^ Specifically, after 5 minutes of rest, participants had 6 BP measurements taken at 1-minute intervals while unattended, and the last 5 measurements were used to calculate the mean SBP and DBP levels.^5^^,^^36^ BP measurements from this technique are highly accurate and reproducible with negligible variability reported between CHMS staff (as expected, because actual BP measurements were conducted in the absence of test personnel).^18^ As was consistent with previous studies, hypertension was defined by a mean SBP ≥ 140 mm Hg or mean DBP ≥ 90 mm Hg (in the absence of diabetes), or a mean SBP ≥ 130 mm Hg or mean DBP ≥ 80 mm Hg (in the presence of diabetes), or if a respondent reported using an antihypertensive medication in the past month.^8^^,^^27^, ^28^, ^29^^,^^37^

#### Medications

Treatment status was then determined by examining self-reported medications taken in the last month. Survey respondents were considered to be using antihypertensive drugs if they reported taking at least 1 of the following medication types based on the Anatomical Therapeutic Chemical (ATC) codes: beta blockers, C07 (excluding C07AA07, C07AA12, and C07AG02); renin-angiotensin system inhibitors, C09; diuretics, C03 (excluding C03BA08); calcium channel blockers, C08; and other antihypertensive drugs, C02 (excluding C02KX01).^8^^,^^27^^,^^28^^,^^30^, ^31^, ^32^ Treatment for diabetes or dyslipidemia was based on a history of taking glucose-lowering medications (A10) or statins (C10AA, C10BA, or C10BX), respectively.

#### Comorbidities

We then determined the presence of selected related comorbidities. Overweight or obesity was defined as a body mass index ≥ 25.0 kg/m^2^ (based on their measured weight and height). Chronic kidney disease was based on an estimated glomerular filtration rate less than 60 mL/min/1.73 m^2^.^38^ Diabetes was defined by self-report, a glycated hemoglobin A1c of ≥ 6.5%, or the use of a glucose-lowering medication. Dyslipidemia was similarly defined by self-report or the use of statins. Finally, a history of heart attack or stroke was based on self-report.

### Recategorization of hypertension

We then estimated the possible effect of inaccurate BP devices that systematically overestimated or underestimated BP on apparent hypertension status. To determine the upper extreme, 5 mm Hg and 10 mm Hg were added to the mean SBP and mean DBP (as measured by the BpTRU devices); similarly, to estimate the lower extreme, 5 mm Hg and 10 mm Hg were subtracted from the mean SBP and mean DBP (as measured by the BpTRU devices), and the prevalence of hypertension was re-estimated using the definition provided here.

### Statistical analysis

We obtained stable population estimates by pooling data from all available cycles of the CHMS.^35^ To account for the complex sampling design, we used the provided survey weights to ensure that the estimates from the analytical sample were representative of the Canadian population. The corresponding variances (95% confidence intervals [CIs] were estimated using bootstrap weights, as recommended by Statistics Canada.^21^, ^22^, ^23^, ^24^, ^25^, ^26^^,^^34^^,^^35^ Descriptive statistics for baseline characteristics, consisting of means and proportions were reported for participants with hypertension. The potential impact of BP overestimation and underestimation was then estimated for the population and according to sex and age strata. Discordance in hypertension status according to differences in SBP and DBP measurements was presented as a reclassification matrix. All statistical analyses were conducted using Stata 16 (StataCorp LLC, College Station, TX) in the Prairie Regional Research Data Centre in Calgary, Alberta, Canada.

## Results

After applying respondent-specific survey weights, there were 25,436,000 Canadian adults between the ages of 20 and 79 years represented during the study period (2007 to 2019), including 6,050,065 (23.8%; 95% CI, 22.5-25.0) who had diagnoses of hypertension based on the presence of high BP or use of antihypertensive drugs (Table 1). The mean age of those with hypertension was 60.4 years (95% CI, 59.9-60.8), with a slight preponderance of male patients (54.8%; 95% CI, 52.6-56.9). The vast majority of individuals with hypertension were overweight or obese (80.3%; 95% CI, 77.7-82.8). Dyslipidemia (52.4%; 95% CI, 50.1-54.7) and diabetes (26.5%; 95% CI, 24.5-28.6) were the most common comorbidities, affecting approximately one-half and one-quarter of people with hypertension, respectively, followed by chronic kidney disease (13.6%; 95% CI, 12.1-15.1), history of heart attack (8.6%; 95% CI, 7.6-9.9), and history of stroke (2.4%; 95% CI, 1.9-3.0).

**Table 1:** Characteristics of adults aged 20 to 79 years with hypertension (N = 6,060,065), 2007 to 2019

Categories	Number represented	Proportion (95% CI)
Total with hypertension	6,050,065	23.8∗ (22.5-25.0)
Mean age, years		60.1
Age bands, %		
20 to 39 years	304,084	5.0 (4.0-6.3)
40 to 59 years	2,309,084	38.2 (35.9-40.5)
60 to 69 years	201,745	33.3 (31.5-35.2)
70 to 79 years	1,419,437	23.5 (0.1-21.8)
Sex		
Men	3,313,372	54.8 (52.6-56.9)
Women	2,736,692	45.2 (43.1-47.4)
Overweight or obese	4,826,414	80.3 (77.7-82.8)
Chronic kidney disease	801,725	13.6 (12.1-15.1)
Diabetes	1,603,596	26.5 (24.5-28.6)
Dyslipidemia	3,170,001	52.4 (50.1-54.7)
Heart attack	520,869	8.6 (7.6-9.9)
Stroke	144,815	2.4 (1.9-2.6)

Presence of hypertension, defined as BP > 140/90 mm Hg for people without diabetes and > 130/80 mm Hg for people with diabetes.

CI, confidence interval.

∗ Proportion of respondents with hypertension based on total number of Canadian adults represented (N = 25,436,000). Values are expressed as percentages or means (95% CI). Percentages, means, and 95% CIs are based on weighted and bootstrapped estimates.

We estimated the proportion of Canadian adults who would be classified as having hypertension to increase to 25.0% (95% CI, 23.7-26.2) and 27.2% (95% CI, 25.8-28.5) if their SBPs were systematically overestimated by 5 mm Hg and 10 mm Hg, respectively (Table 2). The proportion with hypertension was even greater at 28.0 (95% CI, 26.8-29.3) and 36.0 (95% CI, 34.5-37.5) if the DBP was overestimated by 5 mm Hg and 10 mm Hg, respectively. At the extreme, if both SBP and DBP were overestimated 10 mm Hg, nearly one-half of Canadian men (42.9%; 95% CI, 41.0-44.9) and one-third of Canadian women (32.0; 95% CI, 30.1-34.0) would be classified as being hypertensive. Moreover, the prevalence of hypertension would be as high as two-thirds (67.0%; 95% CI, 65.1-68.8) in Canadians aged 60 years and older.

**Table 2:** Potential impact on hypertension prevalence with BP measurement overestimation ranging from 5 to 10 mm Hg

Categories	AOBP from CHMS, % (95% CI)	Only SBP overestimated		Only DBP overestimated		SBP or DBP overestimated	
		by 5 mm Hg	by 10 mm Hg	by 5 mm Hg	by 10 mm Hg	by 5 mm Hg	by 10 mm Hg
Total	23.8 (22.8-25.1)	25.0 (23.7-26.2)	27.2 (25.8-28.5)	28.0 (26.8-29.3)	36.0 (34.5-37.5)	28.8 (27.5-30.1)	37.4 (35.9-38.9)
Sex							
Male	26.3 (24.5-28.2)	27.5 (25.7-29.4)	29.6 (27.8-31.5)	32.6 (30.8-34.5)	41.9 (40.0-43.9)	33.2 (31.3-35.1)	42.9 (41.0-44.9)
Female	21.3 (19.9-22.8)	22.5 (21.0-24.0)	24.7 (23.2-26.3)	23.5 (22.0-25.1)	30.1 (28.2-32.1)	24.5 (23.0-26.1)	32.0 (30.1-34.0)
Age group, years							
20-59	13.5 (12.3-14.9)	14.4 (13.1-15.7)	16.0 (14.7-17.5)	18.7 (17.3-20.1)	27.6 (25.9-29.2)	19.0 (17.6-20.4)	28.2 (35.6-29.9)
60-79	56.7 (54.5-58.8)	59.0 (56.9-61.0)	62.9 (60.8-64.9)	58.0 (55.9-60.2)	62.9 (60.9-64.9)	60.4 (58.3-62.5)	67.0 (65.1-68.8)

AOBP based on measured blood pressure from the CHMS. Hypertension was defined as blood pressure > 140/90 mm Hg (for people without diabetes), blood pressure > 130/80 mm Hg (for people with diabetes), or the use of antihypertensive drugs.

AOBP, automated office blood pressure method; CHMS, Canadian Health Measures Survey; CI, confidence interval; DBP, diastolic blood pressure; SBP, systolic blood pressure.

In contrast, the apparent prevalence of hypertension was less sensitive to underestimation of BP (ie, many individuals with chronic hypertension remained classified as having hypertension because they were still taking antihypertensive drugs). We found that the prevalence of hypertension would be slightly lower at 22.8 (95% CI, 21.6-24.1) and 22.2 (95% CI, 21.0-23.4) if the SBP were underestimated by 5 mm Hg and 10 mm Hg, respectively (Table 3). Likewise, hypertension prevalence would be 22.3 (95% CI, 21.2-23.5) and 21.9 (95% CI, 20.9-23.1) if the DBP were underestimated by 5 mm Hg and 10 mm Hg, respectively. The frequency of hypertension when BP was systematically underestimated (by 5 mm Hg or 10 mm Hg) was broadly similar to the original estimates (based on the actual BpTRU measurements), irrespective of sex or age group.

**Table 3:** Potential impact on hypertension prevalence with BP measurement underestimation ranging from 5 to 10 mm Hg

Categories	AOBP from CHMS, % (95% CI)	Only SBP underestimated		Only DBP underestimated		SBP or DBP underestimated	
		by 5 mm Hg	by 10 mm Hg	by 5 mm Hg	by 10 mm Hg	by 5 mm Hg	by 10 mm Hg
Total	23.8 (22.8-25.1)	22.8 (21.6-24.1)	22.2 (21.0-23.4)	22.3 (21.2-23.5)	21.9 (20.9-23.1)	23.7 (22.5-25.0)	22.9 (21.7-24.2)
Sex							
Men	26.3 (24.5-28.2)	25.5 (23.7-27.4)	25.1 (23.3-27.0)	24.0 (22.5-25.6)	23.6 (22.0-25.2)	26.3 (24.5-28.1)	25.1 (23.4-26.9)
Women	21.3 (19.9-22.8)	20.1 (18.8-21.5)	19.4 (18.2-20.6)	20.6 (19.2-22.1)	20.4 (19.1-21.9)	21.2 (19.8-22.7)	20.8 (19.4-22.2)
Age group, y							
20-59	13.5 (12.3-14.9)	12.9 (11.8-14.2)	12.7 (11.5-14.0)	11.6 (10.8-13.1)	11.6 (10.5-12.7)	13.5 (12.3-14.8)	12.9 (11.7-14.1)
60-79	56.7 (54.5-58.8)	54.4 (52.2-56.5)	52.8 (50.7-54.8)	55.6 (53.4-57.9)	55.4 (53.0-57.7)	56.6 (54.4-58.7)	55.2 (52.5-57.5)

AOBP based on measured blood pressure from CHMS. Hypertension was defined as blood pressure > 140/90 mm Hg (for people without diabetes), blood pressure > 130/80 mm Hg (for people with diabetes), or the use of antihypertensive drugs.

AOBP, automated office blood pressure method; CHMS, Canadian Health Measures Survey; CI, confidence interval; DBP, diastolic blood pressure; SBP, systolic blood pressure.

**Figure 1:**
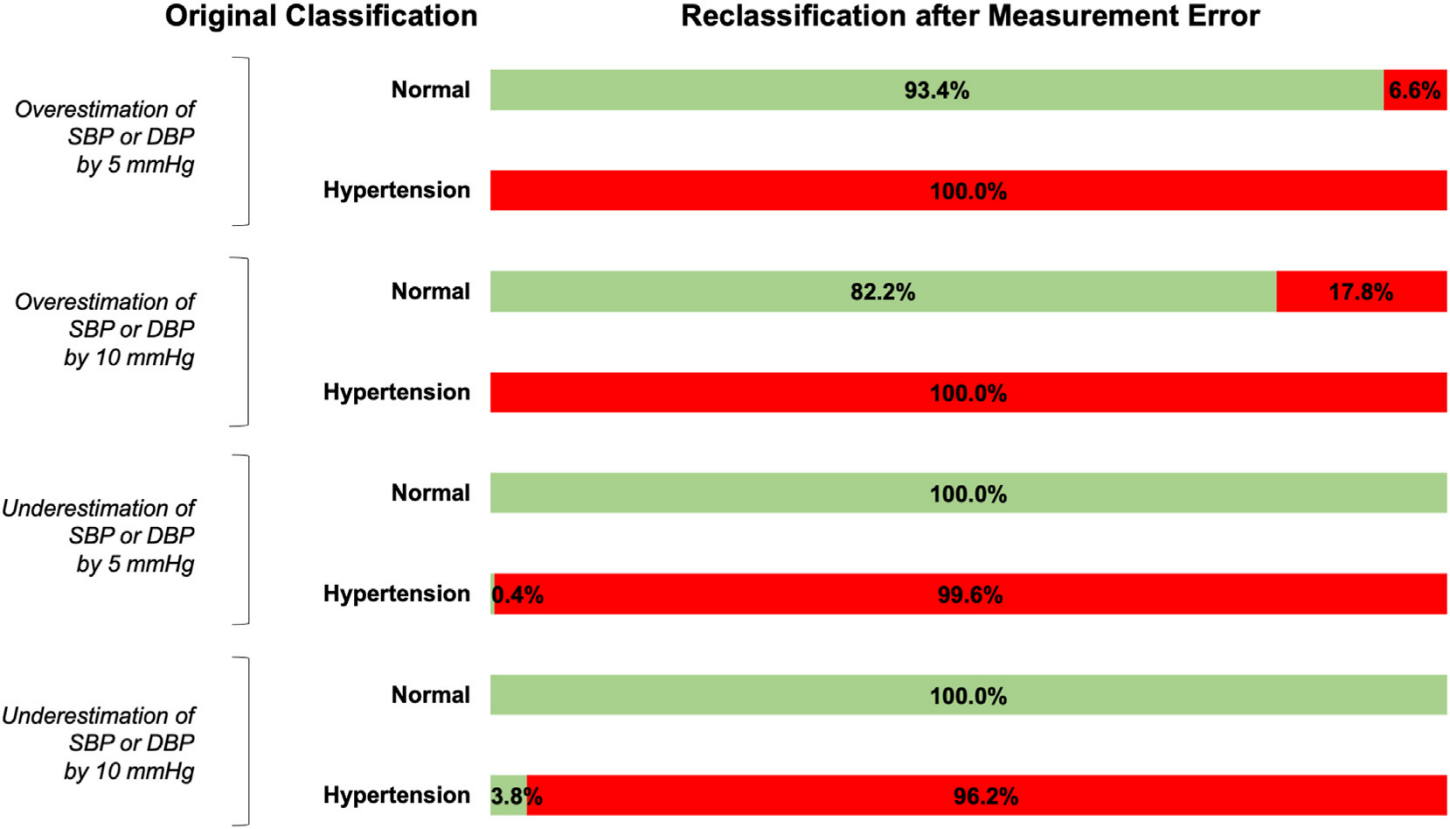
Percentage of people with potential reclassification of caused by systematic overestimation and underestimation of blood pressure, stratified according to actual hypertension status. The percentages represent the proportion of people reclassified with normotension or hypertension per row. Note: the colours of the bars represent the blood pressure category: **green** denotes normotension, and **red** denotes hypertension after reclassification.

We then estimated the number of people potentially reclassified because of changes in BP measurement (Table 4). In absolute terms, when the SBP or DBP was overestimated by 5 mm Hg, there were nearly 1.3 million people (representing 5.0% of the adult Canadian population) who were actually normotensive but potentially reclassified as having hypertension. If SBP or DBP were overestimated by 10 mm Hg, there would be almost 3.5 million people (representing 13.6% of the adult Canadian population) misclassified from being normotensive to hypertensive. In relative terms, among people without any BP disorder, it was estimated that 6.6% and 17.8% would be mislabelled as having hypertension if there were an overestimation of the SBP or DBP by 5 mm Hg or 10 mm Hg, respectively (Fig. 1). On the other hand, when SBP or DBP were underestimated by 5 or 10 mm Hg, comparatively few people with chronic hypertension were falsely reclassified as having normotension (ie, 25,436 and 228,924 people, respectively, each representing less than 1% of the adult Canadian population). In relative terms, if there were systematic underestimation of SBP or DBP by 5 or 10 mm Hg, the percentage of people with hypertension who would be falsely reassured that they had normotension were 0.4% and 3.8%, respectively.

**Table 4:** Reclassification matrix of hypertension status based on number of people represented in Canada

	Original classification using AOBP from the CHMS	
	Normal, number (%)	Hypertension, number (%)
If systematic overestimation of SBP or DBP by 5 mm Hg		
Normal, number (%)	18,110,432 (71.2%)	0 (0%)
Hypertension, number (%)	1,271,800 (5.0%)	6,053,768 (23.8%)
If systematic overestimation of SBP or DBP by 10 mm Hg		
Normal, number (%)	15,922,936 (62.6%)	0 (0%)
Hypertension, number (%)	3,459,296 (13.6%)	6,053,768 (23.8%)
If systematic underestimation of SBP or DBP by 5 mm Hg		
Normal, number (%)	19,382,232 (76.2%)	25,436 (0.1%)
Hypertension, number (%)	0 (0%)	6,028,332 (23.7%)
If systematic underestimation of SBP or DBP by 10 mm Hg		
Normal, number (%)	19,382,232 (76.2%)	228,924 (0.9%)
Hypertension, number (%)	0 (0%)	22.9% (N = 5,824,844)

Total population represented was 25,436,000 Canadian adults. Hypertension was defined as blood pressure > 140/90 mm Hg (for people without diabetes), blood pressure > 130/80 mm Hg (for people with diabetes), or the use of antihypertensive drugs.

AOBP, automated office blood pressure method; CHMS, Canadian Health Measure Survey; CI, confidence interval; DBP, diastolic blood pressure; SBP, systolic blood pressure.

## Discussion

The management of hypertension starts with accurate measurement of BP. Home BP monitoring is recommended by Hypertension Canada as a way to get a better picture of patients’ BP away from the stress of a physician’s office. However, Picone and colleagues^39^ reported that of the 100 best-selling BP devices on Amazon across 10 countries, an average of 79% had no evidence of validation. In Canada, Chan et al.^13^ recently reported that approximately one-half of BP devices are not validated and could be inaccurate. We undertook this study to determine the population health implications of underestimation or overestimation of BP using unvalidated devices. Using Canadian Health Measures Survey data to approximate the Canadian population, we found that overestimation of BP by an unreliable device of only 10 mm Hg would falsely raise the prevalence to approximately 43% in men and 32% in women (relative increases of 63% and 50%, respectively, over actual prevalence), which might result in inappropriate prescription of antihypertensive medications (overtreatment) of approximately 3.5 million Canadians. This signals the need for mandatory validation of BP devices.

Several measures for validation of BP devices existed until the establishment of the universally accepted International Organisation for Standardization Standard (ISO 81060-2:2018) in 2017.^11^^,^^40^ These standards for validation have specific criteria, including a minimum sample size, number of measurements, ranges of BP measurements, and in particular require the average of the difference between the device and the reference standard be less than 5 mm Hg (with the standard deviation being less than 8 mm Hg). This refers to the average difference from a minimum of 85 participants and 255 measurements from a validation study. We chose a similar threshold of 5 and also 10 mm Hg as an arbitrary but clinically meaningful difference, which has also been used in previous studies,^14^^,^^16^

We found that underestimation of BP resulted in little change in classification of hypertension status. This was largely because most respondents who had hypertension are often already treated with an antihypertensive drug (nearly 80%),^8^ so that they would remain classified as having hypertension irrespective of BP measurement. Although BP underestimation would be expected to have large repercussions on national hypertension awareness, treatment, and control, these could not be ascertained here because of the retrospective nature of our study and its cross-sectional design. Underestimation would, however, imply that these individuals would be at higher risk of downstream end organ damage from hypertension than the reported BP value would suggest.

Jones and colleagues^41^ estimated that a systematic measurement error of 5 mm Hg less than true BP would misclassify 21 million Americans as having normal BP, depriving them of the benefits of antihypertensive therapy. In the same study, if a device measured BP that was 5 mm Hg more than true BP, it would misclassify 27 million as hypertensive.^41^ Campbell and colleagues^42^^,^^43^ have reviewed the implications of poor measurement technique and instrumentation, which might even worsen matters. Whether the measurement error is caused by technique or inaccurate devices, the implications are clinically important on both an individual and a population level.

### Strengths and limitations

A strength of our study was that we used high-quality data from the CHMS, which allowed us to quantify the potential effect of BP measurement error using a nationally representative sample of Canadians in which BP was measured using a validated, automated device using a standardized technique. However, our findings must be interpreted in the context of the study design. Our modelling study assumed the worst-case scenario: that is, the most extreme effect that would result if all BP measurements were over- or underestimating BP by 5 or 10 mm Hg. In reality, the absolute number of people reclassified by inaccurate BP measurement would likely be lower. Also, as was consistent with previous studies, we defined hypertension by the presence of high BP or use of antihypertensive drugs using ATC codes.^8^^,^^27^^,^^28^^,^^30^, ^31^, ^32^ Admittedly, however, some medications, such as beta blockers, might have been prescribed for other conditions, potentially leading to some degree of misclassification, although this would have likely been small.^32^ Finally, we were only able to look at the implications of inaccurate BP devices on classification and misclassification of hypertension but not the downstream effects of overtreatment and undertreatment and outcomes. Even so, it would seem reasonable to infer that overdiagnosis would lead to unnecessary pharmacologic treatment in some individuals who would be exposed to potential side effects with little benefit, whereas underestimation of BP may result in false reassurance for others who could benefit from treatment intensification but are now overlooked. Finally, the CHMS response rates were approximately 50%, and it is possible that an unknown bias may have been introduced if nonrespondents were systematically different than those who participated.^21^, ^22^, ^23^, ^24^, ^25^, ^26^ Addressing this, we applied the appropriate survey weights so that the included respondents would be representative of the underlying population of interest, according to sociodemographic characteristics (eg, age and sex).^20^

Hypertension Canada has a Device Recommendation Program that reviews validation data on BP devices (www.hypertension.ca/healthcare-professionals/recommended-devices). However, there is, at present, no requirement for manufacturers to perform validation studies on their devices, and these devices are never submitted to Hypertension Canada for review. BP devices are considered to be low-to-moderate risk, and their approval in most jurisdictions worldwide (including in Canada), and authorities approve then based on the physical safety features rather than accuracy and performance characteristics. Although consumer organizations may have recommendations, these are also often based on cost, ease of use, or appearance rather than accuracy.^11^ Our study has shown that even only 10 mm Hg of overestimation of BP could lead to the misclassification of individuals with normal BP as having hypertension by more than 50%, potentially affecting 3.5 million Canadians. This would likely lead to inappropriate use of antihypertensive medications, adverse effects such as hypotension, and costs to the health care system. As such, until mandatory device validation is enacted by regulatory authorities, clinicians and patients should be cautious in their selection of BP devices and only use Hypertension Canada-recommended devices.

## Conclusions

The proper management of BP begins with accurate measurement. Many home BP devices have not been validated for accuracy, and this may also be true of certain clinic BP devices as well. We found that an overestimation of BP by only 10 mm Hg could potentially misclassify persons with normal BP as having hypertension, potentially leading to inappropriate antihypertensive treatment and associated costs and adverse effects. This highlights the need for regulatory authorities to mandate validation of BP devices before they can be sold. Until then, clinicians and patients should be careful in their selection of BP devices, using the Hypertension Canada Recommended Device Program to guide selection.
